# Supplementary figures and images for: Detection performance of an X-band marine radar system for free-flying Asian particolored bats (Vespertilio sinensis)
Source: PLoS One. 2025 Nov 20;20(11):e0337422. doi: 10.1371/journal.pone.0337422 (PMC12633934; doi:10.1371/journal.pone.0337422)

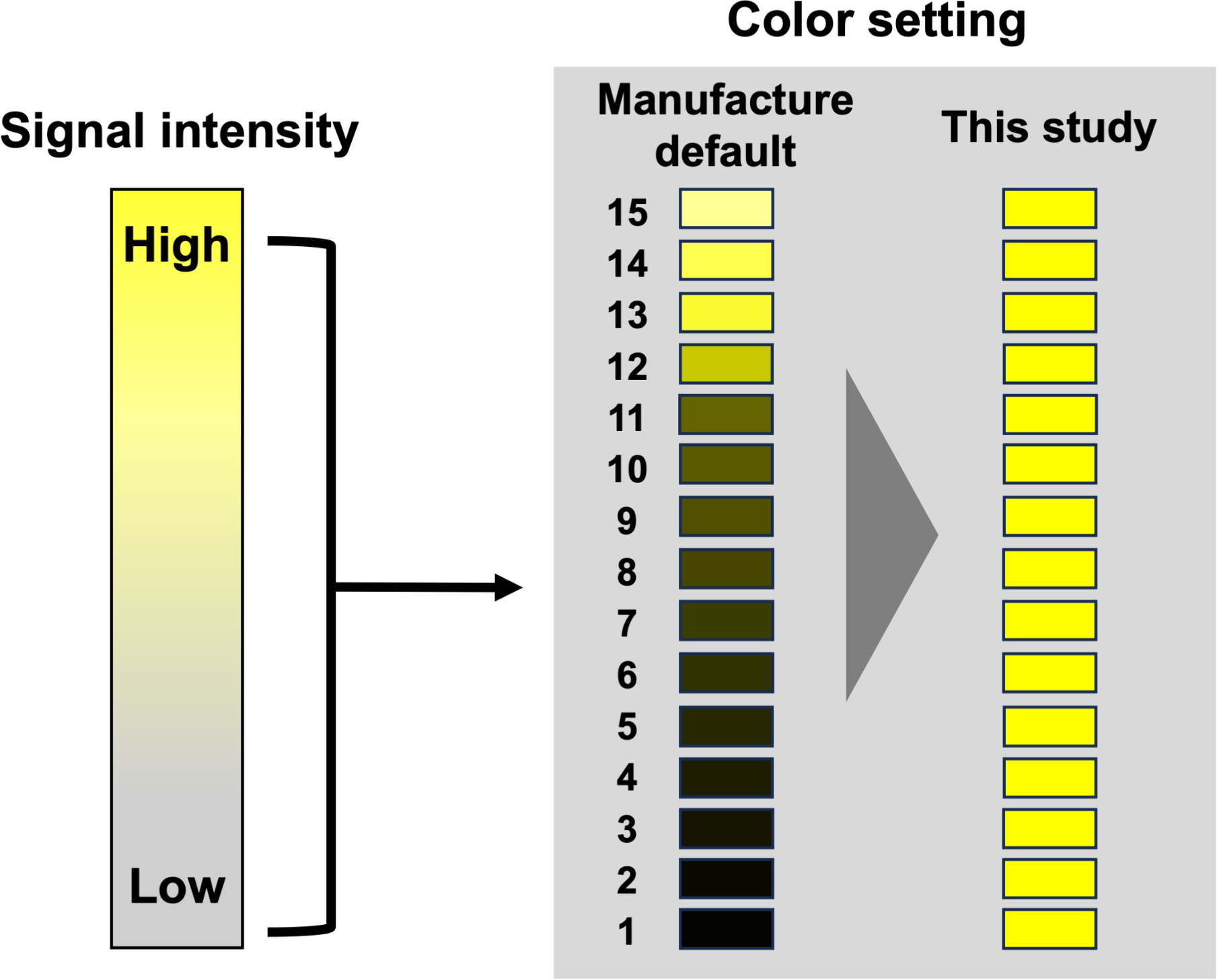

Supplement: S1 Fig — (TIF) [file pone.0337422.s001.tif]

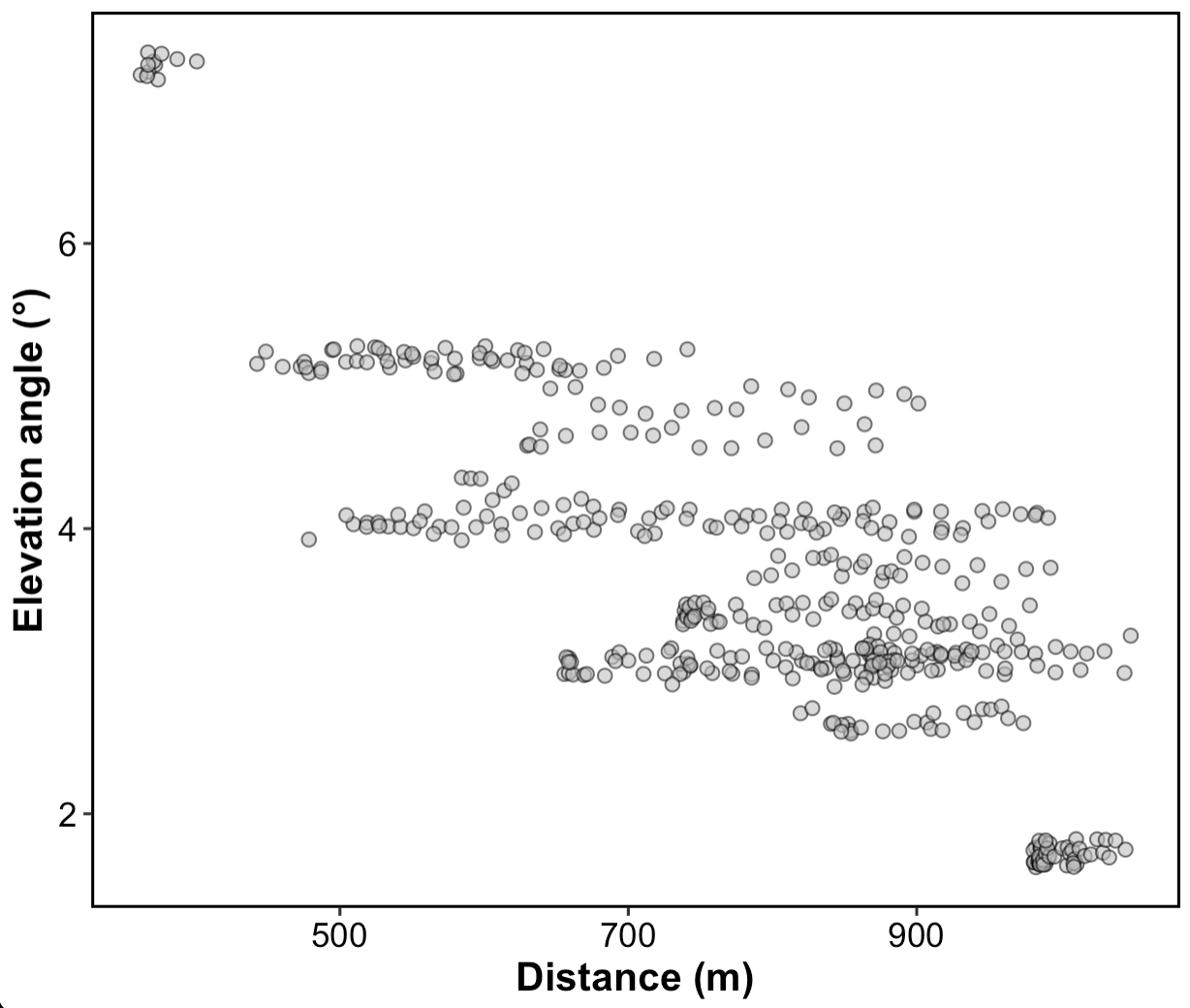

Supplement: S2 Fig — (TIF) [file pone.0337422.s002.tif]

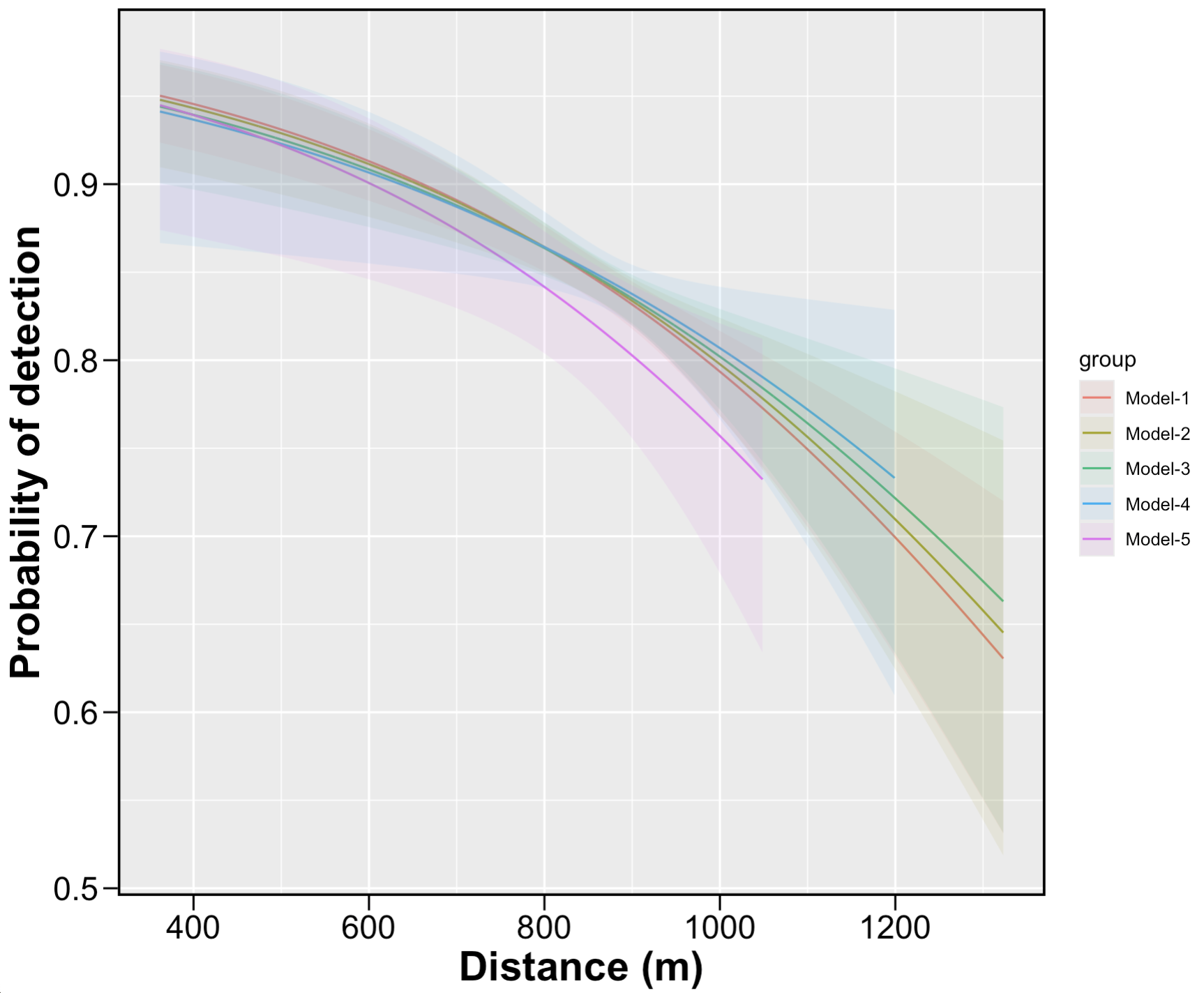

Supplement: S3 Fig — Model parameter estimates for each prediction curve were presented in S6 Table. (TIF) [file pone.0337422.s003.tif]
