# Supplementary material for: Detection performance of an X-band marine radar system for free-flying Asian particolored bats (Vespertilio sinensis)
Source: PLoS One. 2025 Nov 20;20(11):e0337422. doi: 10.1371/journal.pone.0337422 (PMC12633934; doi:10.1371/journal.pone.0337422)
Supplement: S1 File — (DOCX) [file pone.0337422.s010.docx]

**S1 File. Post-hoc power analysis.**

Due to the small number of tracks (n = 25) used in the generalized linear mixed model, we performed a power analysis using the R package simr (Green and MacLeod, 2016), even though extracting the data on bat tracks was already complete. The table below shows the power estimated by varying the number of tracks (Table 1). With the current sample size, the power of the predictor (distance) was 86.0% (95% CI: 83.7−88.1%) indicating an acceptable value compared to the traditional threshold of 80%. We did not include this in the main text because it was a post-hoc power based on results that had already been observed (did not indicate true power). It was not capable of providing new information beyond what was available in the model analysis results.

**Table 1. Power for predictor “distance”.**

| **Number of tracks** | **Power (%)** | **95% CI (lower−upper)** | **Number of observations** |
| --- | --- | --- | --- |
| 5 | 9.3 | 7.6−11.3 | 52 |
| 10 | 25.2 | 22.5−28.0 | 115 |
| 15 | 46.7 | 43.6−49.9 | 193 |
| 20 | 65.1 | 62.1−68.1 | 303 |
| 25 | 86.0 | 83.7−88.1 | 457 |

**Reference**

Green P, MacLeod CJ. SIMR: An R package for power analysis of generalized linear mixed models by simulation. Methods in Ecology and Evolution. 2016; 7(4):493-498. https://doi.org/10.1111/2041-210X.12504
