# Supplementary material for: Detection performance of an X-band marine radar system for free-flying Asian particolored bats (Vespertilio sinensis)
Source: PLoS One. 2025 Nov 20;20(11):e0337422. doi: 10.1371/journal.pone.0337422 (PMC12633934; doi:10.1371/journal.pone.0337422)
Supplement: Attachment — Submitted filename: Response to Reviewers.docx [file pone.0337422.s013.docx]

Manuscript ID: PONE-D-25-24846

Response to Reviewers

Dear Dr. Zhang,

Thank you for giving us the opportunity to submit a revised manuscript “Detection performance of an X-band marine radar system for free-flying Asian particolored bats (*Vespertilio sinensis*)” for publication in PLOS ONE. We wish to express our appreciation to the time and effort that you and the reviewers dedicated to providing feedback on our manuscript and are grateful for the insightful comments on and valuable improvements to our paper. We have incorporated most of the suggestions made by the editor and the reviewers. Please see below the point-by-point author’s responses (written in blue) to the editor and the reviewer’s comments. All page numbers and line numbers refer to the revised manuscript file (Revised Manuscript with Track Changes) with tracked changes.

**Responses to Academic Editor’s comments**

Journal Requirements:

When submitting your revision, we need you to address these additional requirements.

1. Please ensure that your manuscript meets PLOS ONE's style requirements, including those for file naming. The PLOS ONE style templates can be found at

<https://journals.plos.org/plosone/s/file?id=wjVg/PLOSOne_formatting_sample_main_body.pdf> and

<https://journals.plos.org/plosone/s/file?id=ba62/PLOSOne_formatting_sample_title_authors_affiliations.pdf>

<<https://track.editorialmanager.com/CL0/https:%2F%2Fjournals.plos.org%2Fplosone%2Fs%2Ffile%3Fid=ba62%2FPLOSOne_formatting_sample_title_authors_affiliations.pdf/1/010f01975d8ae968-df934054-745d-42bd-92aa-e69ee3247976-000000/UN7YuMcv_zX5H8Cnm2laneJfibSEqZHq8nwVNnfvhRo=215>>

**Author response:** Thank you for your guidance. We have reformatted the manuscript according to the above style guidelines. Furthermore, an additional affiliation information of the authors on the title page have been added to ensure continuity in communication about the manuscript (p. 1, lines 14). The reason is that the institutional address provided will soon be deactivated due to a change in the author’s affiliation. Additionally, in accordance with the reviewer’s (Reviewer 2) suggestion, we have added the species name to the title of our manuscript. The revised title is as follows:

[p. 1, lines 4-6] “Detection performance of an X-band marine radar system for free-flying Asian particolored bats (*Vespertilio sinensis*)”

2. In your Methods section, please provide additional information regarding the permits you obtained for the work. Please ensure you have included the full name of the authority that approved the field site access and, if no permits were required, a brief statement explaining why.

**Author response:** Thank you for your comment. We have added the following text to the Methods section:

[p. 4, lines 97-98] “Our field work was approved by the land manager, the Land Improvement District of Nishikanbara (Permit Number 486).”

3. We note that Figure 1 in your submission contain [map/satellite] images which may be copyrighted. All PLOS content is published under the Creative Commons Attribution License (CC BY 4.0), which means that the manuscript, images, and Supporting Information files will be freely available online, and any third party is permitted to access, download, copy, distribute, and use these materials in any way, even commercially, with proper attribution. For these reasons, we cannot publish previously copyrighted maps or satellite images created using proprietary data, such as Google software (Google Maps, Street View, and Earth). For more information, see our copyright guidelines: <http://journals.plos.org/plosone/s/licenses-and-copyright>.

We require you to either (1) present written permission from the copyright holder to publish these figures specifically under the CC BY 4.0 license, or (2) remove the figures from your submission:

A. You may seek permission from the original copyright holder of Figure 1 to publish the content specifically under the CC BY 4.0 license.

We recommend that you contact the original copyright holder with the Content Permission Form
(<http://journals.plos.org/plosone/s/file?id=7c09/content-permission-form.pdf>) and the following text: “I request permission for the open-access journal PLOS ONE to publish XXX under the Creative Commons Attribution License (CCAL) CC BY 4.0 (<http://creativecommons.org/licenses/by/4.0/>). Please be aware that this license allows unrestricted use and distribution, even commercially, by third parties. Please reply and provide explicit written permission to publish XXX under a CC BY license and complete the attached form.”

Please upload the completed Content Permission Form or other proof of granted permissions as an ""Other"" file with your submission.

In the figure caption of the copyrighted figure, please include the following text: “Reprinted from [ref] under a CC BY license, with permission from [name of publisher], original copyright [original copyright year].”

B. If you are unable to obtain permission from the original copyright holder to publish these figures under the CC BY 4.0 license or if the copyright holder’s requirements are incompatible with the CC BY 4.0 license, please either i) remove the figure or ii) supply a replacement figure that complies with the CC BY 4.0 license. Please check copyright information on all replacement figures and update the figure caption with source information. If applicable, please specify in the figure caption text when a figure is similar but not identical to the original image and is therefore for illustrative purposes only.

The following resources for replacing copyrighted map figures may be helpful:

USGS National Map Viewer (public domain):

<http://viewer.nationalmap.gov/viewer/>

The Gateway to Astronaut Photography of Earth (public domain):

<http://eol.jsc.nasa.gov/sseop/clickmap/>

Maps at the CIA (public domain):

<https://www.cia.gov/library/publications/the-world-factbook/index.html>

and <https://www.cia.gov/library/publications/cia-maps-publications/index.html>

NASA Earth Observatory (public domain):

<http://earthobservatory.nasa.gov/>

Landsat:

<http://landsat.visibleearth.nasa.gov/>

USGS EROS (Earth Resources Observatory and Science (EROS) Center) (public domain):

[http://eros.usgs.gov/#](http://eros.usgs.gov/)

Natural Earth (public domain):

<http://www.naturalearthdata.com/>

<<http://track.editorialmanager.com/CL0/http:%2F%2Fwww.naturalearthdata.com%2F/1/010f01975d8ae968-df934054-745d-42bd-92aa-e69ee3247976-000000/2FBiXldHDaepEFK-Tlk3t_4d0tvqEd0DnKi5dBZIRdk=215>>

**Author response:** Thank you for your kind guidance. We have removed the map in Figure 1a and replaced it with a map available under permission from the original copyright holder (The Japan Aerospace Exploration Agency). We have updated Figure 1 and its caption with source information. Additionally, we have uploaded the Content Permission Form. We have added the following text to the Figure caption:

[p. 5, lines 101-103] “Basemap reprinted from High-Resolution Land Use and Land Cover Map (Japan) Version 25.04, under a CC BY license, with permission from Earth Observation Research Center (EORC), The Japan Aerospace Exploration Agency (JAXA) (dated 17 July 2025), original copyright 2025.”

**Author’s responses to Reviewer’s comments**

**5. Review Comments to the Author**

**Reviewer #1**

While the study presents an interesting application of marine radar for bat detection, the manuscript contains several critical limitations that compromise its scientific rigor and broader applicability. The methodology lacks robustness in key areas, the data analysis is insufficiently detailed, and the conclusions are overstated given the narrow scope of the research. The paper requires major revisions to address these shortcomings before it can be considered for publication.

**Author response:** We would like to thank you for your insightful comments. These comments have helped us to improve the quality of our paper. We are grateful for the time and energy you expended on our behalf. In the following sections, you will find our responses to each of your comments and suggestions. We hope our revisions address the concerns you have raised. All page numbers and line numbers refer to the revised manuscript file (Revised Manuscript with Track Changes) with tracked changes.

1. The study was conducted at a single site with flat terrain and a specific bat species (Vespertilio sinensis), yet the conclusions imply broad applicability to "quantitative bat surveys over extensive spatial areas." The authors fail to acknowledge how radar performance may vary in complex landscapes (e.g., urban environments, mountainous terrain) or with species of different size/flight behavior. Without replicate experiments in diverse environments or comparisons with other bat species, the findings are inherently site-specific and cannot support the claimed generality.

**Author response:** We appreciate the reviewer’s comment on this point. In accordance with your comments, we have removed the texts of the Conclusions section (p. 16, lines 393-395). To make the study’s limitations clearer, we have also added a new subsection titled “Limitations” to the Discussion (p. 16, lines 375-383). In the Limitations subsection, we have mentioned the need to verify factors affecting radar performance that have not been tested in this study including landscape features and interspecific differences in size or flight behavior. Additionally, for clarification, we have divided the Discussion section into three subsections: Bat detection performance (p. 12, lines 270-323), Ground clutter reduction (p. 14, lines 325-349), and Limitations (p. 16, lines 376-384).

2. The manuscript provides insufficient detail on how radar echoes were differentiated from non-bat targets (e.g., birds, insects, noise). While ornithodolite tracking was used to validate 25 tracks, the vast majority of radar echoes (386 tracks) were manually classified as "potential bat-like characteristics" without explicit criteria (e.g., velocity thresholds, flight patterns). This subjective approach introduces bias and undermines the reliability of the dataset. Additionally, the absence of machine learning-based classification or Doppler signature analysis—common in modern radar studies—limits the methodological rigor.

**Author response:** We appreciate the reviewer’s feedback regarding radar echo extraction procedures. In accordance with the reviewer’s comments, we have added further explanation for this point. While there is the possibility that the observed radar echoes may contain birds and insects, we believe most of the tracks extracted through manual tracking are from bats. To clarify, we have added the following texts to the Bat echo extraction subsection (p. 8-10, lines 184-228). The added texts read as follows:

[p. 8, lines 189-192] “Bird species observed in this area at dusk (grey heron, great white egret, and carrion crow) were generally scarce and only occasionally appeared within the radar observation area. In order to minimize contamination from bird movements, we focused only on echoes that emerged from the area around the locations of bat roosts (viaduct) and moved westward or southwestward. These were the major directions of the movement of bats observed in our study site.”

[p. 9, lines 203-213] “Furthermore, we examined whether the movement speed of the obtained radar tracks overlapped with that of bats rather than birds or insects. For the radar tracks, the mean ground-speed was 10.4 m/s (range = 4.2–16.5 m/s), suggesting that the majority of the tracked echoes were flying vertebrates since the typical flight speed of insects is less than 5 m/s [37, 38]. This data was consistent with the ground-speed of Asian particolored bats (mean = 9.9 m/s, range = 5.4–16.4 m/s) recorded by the ornithodolite. For birds observed in the same study area, the mean ground-speeds of grey herons, great white egrets, and carrion crows recorded by the ornithodolite were 25.0 m/s (range = 12.3–37.7 m/s, n = 13), 21.5 m/s (range = 10.6–37.9 m/s, n = 13), and 23.4 m/s (range = 14.4–30.2 m/s, n = 6), respectively. The ground-speed of the radar tracks was not significantly different from that of Asian particolored bats, but was significantly slower than that of birds (Steel-Dwass test: radar tracks vs. bats, W = −2.557, p = 0.348, radar tracks vs. grey herons, W = 8.441, p < 0.001, radar tracks vs. great white egrets, W = 7.943, p < 0.001, radar tracks vs. carrion crows, W = 5.880, p < 0.001). Therefore, we assumed that the manual tracking data were rarely contaminated by birds or insects.”

As the reviewer suggested, we agree that applying machine learning-based classification or Doppler signature analysis of radar echoes would be valuable. Regrettably, however, we are unable to do both because we lack sufficient training data for bats and birds, and the radar used in this study lacks a system for utilizing radial velocity. We are now collecting radar data of bats and birds for discriminating targets based on machine learning. Therefore, we would like to achieve this point in future research.

3. The analysis relies on only 25 validated bat tracks, which is statistically insufficient to robustly model detection probability as a function of distance. The generalized linear mixed model (GLMM) may lack power to detect subtle effects, and the high proportion of non-detection events (75 instances) could skew results. The authors do not report effect sizes (e.g., odds ratios) or conduct sensitivity analyses to assess how sample size impacts model stability. Without larger datasets or cross-validation, the claimed "70% detection probability at 1.0 km" is questionable.

**Author response:** We appreciate the reviewer’s comment. In accordance with your comment, we have added the odds ratio of the predictor (distance) to the Results section (p. 11, lines 257-258) and Table 2 (p. 11, lines 265-266). We have also revised the text describing the results of the analysis in the Results section as follows:

[p. 11, lines 256-258] “The distance showed a weak but statistically significant negative effect on the probability of detection (GLMM: odds ratio = 0.997, 95% confidence interval: 0.995−0.999, Table 2).”

Additionally, we have added the following text on the limitations of our sample size to the Discussion section:

[p. 12-13, lines 287-292] “Distance exhibited a significant effect on POD, but the estimated effect size was small. This may be due to the limited sample size of the identified bat tracks (n = 25), which could lead to bias in the model estimates. Although a larger dataset is needed for robust modeling, it is worth noting that bat tracks consisted of a high proportion of detected echoes. The observed proportion of detection events per track ranged from 63% to 100% (mean = 83%), suggesting limited variation in POD. This may be the reason why the effect size of distance was estimated to be small. It should be noted that our results were obtained solely within this study’s settings and observation range.”

Furthermore, in response to the reviewer’s concern, we have performed a power analysis using the R package simr (Green and MacLeod, 2016) with the current sample size although extracting the data on bat tracks had already been completed. The power of the predictor was 86.0% (95% CI: 83.7−88.1%), indicating an acceptable value compared to the traditional threshold of 80%. However, we did not include this in the manuscript because it was a post-hoc power based on results that had already been observed (did not indicate true power). It was not capable of providing new information beyond what was available in the model analysis results.

4. The study focuses solely on distance as a predictor of detection probability but overlooks other critical factors. Bat flight altitude, for instance, could influence signal strength given the radar’s 22° vertical beam width, yet the authors do not analyze elevation angle data from the ornithodolite. Additionally, the manuscript fails to report environmental parameters (e.g., rain, fog) during data collection, despite known radar performance degradation in such conditions. Furthermore, the study only observes bats in straight-line post-roost flight, ignoring foraging behaviors like erratic maneuvers that may affect detectability. These omissions limit the model’s ecological relevance and its ability to generalize to real-world bat movement patterns.

**Author response 1 regarding effect of elevation angle:** We appreciate the reviewer’s suggestion regarding this point. We agree that this is an important point. However, we believe it appropriate to use only distance in our model analysis for the following reasons. First, we have confirmed that there was a co-linearity between distance and elevation angle (Spearman’s rank correlation: r^2^ = −0.71, p < 0.001). To explain why we did not include elevation angle in the model analysis, we have added the following text to the Statistical analysis subsection:

[p. 10, lines 231-235] “The purpose of our analysis was to verify the effect of distance on the probability of bat echo detection. However, in addition to distance, bat flight altitude (elevation angle from antenna) also affects radar detectability. We checked for co-linearity between two variables: distance and elevation angle (recorded by the ornithodolite) from the radar position. Since a negative correlation was observed between distance and elevation angle (Spearman’s rank correlation: r^2^ = –0.71, p < 0.001), we only used distance in the following model analysis.”

Further, we have also analyzed the effect of elevation angle on bat echo detection using a simple generalized linear mixed model (GLMM) with elevation angle as the explanatory variable and track ID as the random factor. However, elevation angle showed no significant effect on probability of detection (GLMM: Odds ratio = 1.19, 95% CI: 0.89−1.60, z = 1.17, p = 0.24). For clarification, we have added the following text to the Discussion section regarding the effect of bat flight altitude on radar detectability:

[p. 13, lines 299-305] “Two factors require further verification. First, the flight altitude of bats could influence the detection capability of radar. In general, the signal power returned from a target decreases as the elevation angle from the beam center increases. In our study, the mean elevation angle from the radar position to the bat positions recorded by the ornithodolite was 3.1° (range = 0.9–8.7°). It can be assumed that bat echoes used in model analysis were detected within the main lobe (± 11° from the beam center) of the vertical beam axis, suggesting a relatively weak effect on detection probability. However, it should be noted that the radar detectability of bats flying at higher altitudes may not be consistent with the results of this study. This is due to the fact that we did not include elevation angle in the model analysis to avoid the issue of co-linearity.”

**Author response 2 regarding conditions during data collection:** Thank you for pointing this out. Our radar observation was conducted under conditions without rainfall or fog. We have added environmental conditions during data collection to the Methods section as follows:

[p. 7, lines 144] “Radar surveys were performed for a total of five days in early July 2023 under conditions with no rainfall or fog, both of which affect radar detection performance.”

**Author response 3 regarding effect of bat behaviors:** We appreciate the reviewer’s comment on this point. We agree that this point requires clarification. While we recognize that bat flight behaviors would affect radar detectability, tracking bat’s erratic movements by ornithodolite was technically difficult. For this reason, we focused our ornithodolite tracking efforts on flights soon after bats emerged from roosts at dusk. We have added the following text about the technical limitations of direct observation using the ornithodolite to the Discussion section:

[p. 14, lines 321-323] “Moreover, it was technically difficult to track the foraging behaviors of bats due to their erratic maneuvers. For these reasons, additional techniques are required to accurately identify species in complete darkness and track more complex behaviors.”

Additionally, we have added the following text regarding the effect of bat’s behaviors on radar detectability to the Discussion section:

[p. 13, lines 305-312] “Second, variations in bat flight behavior may also affect radar detectability. For the purpose of accurate target identification, we focused our radar observations at dusk. Observed bats were in the transition phase after emerging from their roosts and exhibited straight flight paths. Therefore, we could not verify detectability of bats during foraging behavior, which involves more complex flight paths. It has been previously documented that for aging bats exhibit erratic flight patterns [40, 41, 42]. The flight tortuosity of bats may influence successful detection and tracking, as has been suggested by previous avian radar studies [29, 30]. At this time, the impact of behavioral irregularities on radar detectability remains uncertain. It is imperative to accumulate data on radar performance for behavioral variations both within and between bat species in future research endeavors.”

5. The authors claim radar "circumvents range limitations" of acoustic surveys but do not directly compare radar detections with simultaneous acoustic data (e.g., bat pass rates) within the same spatial domain. Without such comparisons, it is impossible to quantify the added value of radar or validate its superiority in real-world scenarios. This missed opportunity weakens the study’s novelty and practical implications.

**Author response:** We appreciate the reviewer’s feedback. We agree that additional information on comparisons between radar and acoustic devices would be valuable. However, if the reviewer is referring to the difference in capability to quantify bat activity, then we believe that directly comparing data between the two methods would not be possible. This is because acoustic data obtained by a bat detector cannot reflect the actual number of individuals passing through the detection area. Specifically, acoustic bat detectors cannot distinguish between situations in which a single bat is repeatedly detected and situations in which multiple bats pass by. On the other hand, if the reviewer is stating the difference in detectable range between radar and a bat detector, then we believe this point can be addressed by adding a discussion of information from existing literature. Thus, we have added the following text to the Discussion section:

[p. 13, lines 293-298] “The maximum detection distance recorded in this study (1,048 m) was much greater than that of a single acoustic detector (approximately 40 m) for bats emitting low-frequency calls, as shown in previous literature [23, 24]. This may represent an advantage of marine radar for monitoring bat movements over large areas. However, radar’s horizontal detection range is highly dependent on landscape features that cause ground clutter. Furthermore, the detectable range for smaller species of bats is likely to be shorter. To clearly demonstrate the superiority of the radar, it is necessary to perform comparable validations in conjunction with acoustic devices or species of varying sizes.”

Furthermore, we have also revised the relevant text in the Introduction section as follows:

[p. 3, lines 60-61] “The utilization of marine radar for the study of bats holds the potential to enable the detection and tracking of bat movements over large spatial areas [23, 24].”

6. While lowering the antenna height reduced ground clutter, the authors do not theoretically justify this approach (e.g., using radar propagation models to explain how vegetation acts as a "clutter fence"). The analysis also fails to address trade-offs, such as reduced coverage at low antenna heights due to Earth’s curvature. A more rigorous discussion should link empirical results to radar theory and cite prior studies on clutter mitigation in avian radar.

**Author response:** We agree that this point requires further discussion. For clarification, we have created a new subsection titled “Ground clutter reduction” in the Discussion section (p. 14, lines 325-349). In accordance with the reviewer’s comment, we have added the following text to this subsection regarding how vegetation contributed to reducing ground clutter:

[p. 14, lines 328-341] “Our results suggest that the densely planted rice in the study area may have shielded the lower part of the vertical beam when the antenna height was lowered. A similar effect caused by natural objects, such as trees, has been mentioned in previous literature [16, 19]. Prior studies have shown that the dense foliage of vegetation scatters and attenuates a radar beam’s radiated power as it propagates through the vegetation [43, 44, 45]. This reduces the amount of the reflected signal that returns to the radar receiver. The same process may have reduced the backscattering from the rice fields, thereby decreasing ground clutter.

Historically, previous radar studies have attempted to mitigate ground clutter through a variety of ways. These methods include the installation of clutter shielding fences [16, 23] and the adjustment of antenna angles relative to the ground [24]. Additionally, dedicated avian radar systems have also implemented image processing algorithms that utilize raw reflectivity and/or velocity data to discriminate targets and ground clutter [46, 47]. Although our adopted method has not been widely used, the effect of adjusting the antenna height on ground clutter in radar images has been demonstrated using a commercial marine radar [23]. For a portable commercial marine radar, adjusting the height of the antenna may be a simple option for achieving a larger detectable area.”

Furthermore, we agree that additional discussion is needed on the reduced radar coverage that would result from lowering the antenna height, as the reviewer suggested. We are now investigating this point and intend to report it in a later paper. In response to the reviewer’s comment, we have added the following text to the Ground clutter reduction subsection as an issue for future research:

[p. 14-15, lines 342-349] “Although ground clutter was successfully reduced in this study, we could not rule out the possibility that lowering the antenna height decreased radar coverage near the ground. This phenomenon has been shown by a simulation-based study that modeled radar vertical beam patterns with clutter-shielding fence [23]. In that case, the received signal power from near the ground level (lower altitude) decreased with distance when the fence was installed. Since our study could not determine how rice field vegetation scattered and diffracted the radar beam, the issue of trade-offs between ground clutter reduction and detectable area is a matter for future verification. Furthermore, our performance evaluation was conducted at a single site with flat terrain. Given the highly site-specific nature of ground clutter within radar coverage, the methodology presented here should be subjected to testing at other locations to ascertain its applicability.”

**Reviewer #2**

This manuscript presents a well-designed, field-based study validating the performance of a commercial X-band marine radar (PONE-D-25-24846) for detecting free-flying Asian particolored bats (Vespertilio sinensis). The authors integrate ornithodolite-based tracking to match radar echoes with bat flight trajectories, analyze the impact of antenna height on clutter, and model the probability of detection (POD) using GLMM. This study contributes novel, empirical evidence to a field lacking rigorous performance evaluations of radar systems for bat monitoring and offers practical guidance on range limitations, clutter reduction, and signal processing.

**Reviewer comments:**

Strengths:

Novelty and Relevance: Directly addresses a gap in validating marine radar detection capabilities for bats. High relevance for environmental impact assessments (EIA) and wind energy–wildlife conflict mitigation.

Methodological Rigor: Controlled validation using ornithodolite data for ground truth. Inclusion of clutter reduction, manual echo tracking, and statistical modeling (GLMM) for robust analysis.

Clarity and Detail: The manuscript is well-structured with detailed explanations of radar configuration, antenna adjustment, and data processing steps. Clear and appropriate use of figures (especially Fig 3 and Fig 5).

Practical Implications: Findings inform best practices for radar placement and system settings. Suggests radar applicability up to 1.0 km with >70% POD.

**Author response:** We wish to thank you for your valuable comments and suggestions on our paper. These have greatly helped us to improve the quality of our manuscript. We are very grateful that our paper has been recognized as addressing a gap in validating radar detection performance for bats. In the following sections, you will find our responses to each of your comments and suggestions. All page numbers and line numbers refer to the revised manuscript file (Revised Manuscript with Track Changes) with tracked changes.

**Major Points for Consideration and Improvement:**

1. Add a paragraph in the Discussion explicitly acknowledging the limitations of the sample size and the need for multi-site validation.

**Author response:** We appreciate the reviewer’s comment on this point. In accordance with the comment, we have added a subsection titled “Limitations” (p. 16, lines 376-384) in the Discussion acknowledging the limitations of this study. Furthermore, for clarification, we have also revised the Discussion section into three subsections: Bat detection performance (p. 12, lines 270-323), Ground clutter reduction (p. 14, lines 325-349), and Limitations (p. 16, lines 376-384). The reason is that Reviewer 1 also made similar comments about this study’s limitations. The added paragraph reads as follows:

[p. 16, lines 376-384] “This study has potential limitations. The first shortcoming is the limited sample size of radar tracks that were successfully verified as bat flights (n = 25). It is important to note that the relatively high POD of bat echoes can be achieved within the settings and observation range of this study. A larger dataset would be required for more robust analysis. The second limitation is its focus on a single location and a specific bat species. The detection capability of marine radar may be affected by differences in landscape features, the size of the target species, and the complexity of its behavior. In order to enhance the generalizability and applicability of our findings, it is necessary to validate the radar performance in multiple sites, in more complex terrain, and with other bat species of different size or flight behavior. To achieve this, we need to develop a technique for collecting ground truth data applicable to nighttime and complex behaviors.”

2. Include explicit reporting of CI ranges in the Results text and briefly interpret the slope estimate of the distance variable in practical terms.

**Author response:** In accordance with your comment, we have added the following text in the Results section:

[p. 11, lines 256-259] “The distance showed a weak but statistically significant negative effect on the probability of detection (GLMM: odds ratio = 0.997, 95% confidence interval: 0.995−0.999). This indicates that the proportion of detection events per track decreases with distance from the radar.”

We have also added the odds ratio to Table 2 (p. 11, lines 265-266).

3. Adopt journal-standard reporting conventions for P-values (e.g., p < .001), include effect size estimates where possible, and always identify the test used (e.g., likelihood ratio test).

**Author response:** Thank you for pointing this out. We have followed the journal’s reporting rules for stating p-values and identified the statistical tests in the Methods and Results sections. We have also included effect size estimates in the Results section. The following revisions have been made:

[p. 9, lines 210-212] “The ground-speed of the radar tracks was not significantly different from that of Asian particolored bats, but was significantly slower than that of birds (Steel-Dwass test: radar tracks vs. bats, W = –2.557, p = 0.348, radar tracks vs. grey herons, W = 8.441, p < 0.001, radar tracks vs. great white egrets, W = 7.943, p < 0.001, radar tracks vs. carrion crows, W = 5.880, p < 0.001).” This text has been added in response to comments from Reviewer 1.

[p. 10, lines 233-234] “Since a negative correlation was observed between distance and elevation angle (Spearman’s rank correlation: *r*^2^ = –0.71, p < 0.001), we only used distance in the following model analysis.” This text has also been added in response to comments from Reviewer 1.

[p. 11, lines 256-258] “The distance showed a weak but statistically significant negative effect on the probability of detection (GLMM: odds ratio = 0.997, 95% confidence interval: 0.995−0.999, Table 2).”

[p. 11, lines 259-260] “Additionally, the model with distance was significantly different from the null model (likelihood ratio test: χ^2^ = 7.37, df = 1, p = 0.006).”

4. If no permit was needed for observational tracking, add a clarification (e.g., “No animal handling was conducted, so no ethics permit was required”).

**Author response:** In accordance with the reviewer’s suggestion, we have added the following text to the Methods section:

[p. 8, lines 181-182] “In this study, no animal handling was conducted, so no ethics permission was required.”

5. Consider uploading S1–S4 Tables and corresponding radar images/trajectory files to a public repository for long-term access.

**Author response:** Thank you for your suggestion. We have registered our data including radar images in the Tokushima University data repository (<https://tokushima-u.repo.nii.ac.jp/records/2013473>). The dataset will be assigned a DOI once it is registered in the repository and will be accessible. Due to changes in the author’s affiliation, data registration procedures have been delayed. The following DOI link will be made public in September: <https://doi.org/10.15000/0002013473>

**Minor Suggestions:**

1. Title: Consider specifying the species (Vespertilio sinensis) in the title to emphasize the study’s biological focus.

**Author response:** Thank you for this suggestion. We have added the species name to the title. The revised title reads as follows:

[p. 1, lines 5-6] “Detection performance of an X-band marine radar system for free-flying Asian particolored bats (*Vespertilio sinensis*)”

2. Abstract: Mention the sample size of matched tracks (n = 25) to set expectations early.

**Author response:** In accordance with the reviewer’s suggestion, we have added the sample size to the Abstract as follows:

[p. 2, lines 32] “Following procedure, we identified 25 radar tracks as bat flights.”

3. Terminology: Use consistent terms for "ground clutter reduction" and "clutter fences" for clarity.

**Author response:** We appreciate the reviewer’s suggestion. We have used “ground clutter reduction” instead of “clutter fences” and revised the relevant terms in the Methods section (p. 7, lines 148-159). The revised text is as follows:

[p. 7, lines 150-151] “Although the appearance of ground clutter is inevitable, objects surrounding the radar, such as low vegetation, hills, and man-made structures, can be used to reduce ground clutter [1, 19].”

Additionally, we have renamed the subsection “Clutter reduction” to “Ground clutter reduction” (p. 7, line 148).

4. Typo Fixes: Line 186: "align with that of the rice fields" → consider rephrasing for clarity.

**Author response:** Thank you for pointing this out. We have rephrased the corresponding sentence. The revised text reads as follows on:

[p. 7, lines 153-155] “We expected that matching the height of the radar antenna to the height of the vegetation would reduce clutter. The reason was that the nearest vegetation from the antenna was expected to shield the lower half of the emitted radar beam, as mentioned in the existing literature [16].”

Recommendation:

✅ Accept with Minor Revisions

This is a well-executed, methodologically sound study with high practical utility and scientific value. The improvements suggested above would enhance clarity, reproducibility, and scientific rigor, but do not detract from the manuscript’s core contributions.

Thank you once again for the valuable comments and suggestions made by the Academic Editor and the Reviewers. We hope that the revisions we made are sufficient to make our manuscript suitable for publication in PLOS ONE.

Sincerely,

Yoichi Kawaguchi

Graduate School of Technology, Industrial, and Social Sciences, Tokushima University

2-1 Minamijosanjima-cho, Tokushima, Japan

TEL: +81-88-656-9025

E-mail: kawaguchi@ce.tokushima-u.ac.jp
