# Supplementary material for: Detection performance of an X-band marine radar system for free-flying Asian particolored bats (Vespertilio sinensis)
Source: PLoS One. 2025 Nov 20;20(11):e0337422. doi: 10.1371/journal.pone.0337422 (PMC12633934; doi:10.1371/journal.pone.0337422)
Supplement: Attachment — Submitted filename: Response_to_Reviewers_auresp_2.docx [file pone.0337422.s015.docx]

Manuscript ID: PONE-D-25-24846

Response to Academic Editor and Reviewers

Dear Dr. Zhang,

We would like to thank you for allowing us to resubmit a revised manuscript “Detection performance of an X-band marine radar system for free-flying Asian particolored bats (*Vespertilio sinensis*)” for publication in PLOS ONE. We appreciate the time and effort that you and the reviewers dedicated to providing comments on our paper. We have incorporated the comments and suggestions made by the reviewer. On the following pages, we present our point-by-point responses to the comments (written in blue). All page numbers and line numbers refer to the revised manuscript file (Revised Manuscript with Track Changes) with tracked changes.

**Responses to Academic Editor’s comments**

**Journal Requirements:**

If the reviewer comments include a recommendation to cite specific previously published works, please review and evaluate these publications to determine whether they are relevant and should be cited. There is no requirement to cite these works unless the editor has indicated otherwise.

Please review your reference list to ensure that it is complete and correct. If you have cited papers that have been retracted, please include the rationale for doing so in the manuscript text, or remove these references and replace them with relevant current references. Any changes to the reference list should be mentioned in the rebuttal letter that accompanies your revised manuscript. If you need to cite a retracted article, indicate the article’s retracted status in the References list and also include a citation and full reference for the retraction notice.

**Author response**: Thank you for your comments. We apologize for not mentioning the addition of references in the previous rebuttal letter. In the previous revision process, we have carefully considered the reviewer’s comments (reviewer #1, comments #2 and #6) and decided to cite the following articles (Ref. 37, Ref. 43-47). We believe these publications were necessary in order to justify our bat’s echo extraction procedures and to enable more rigorous discussions about ground clutter reduction. Additionally, we have removed a publication (Appelhans T, Detsch F, Reudenbach C, Woellauer S. Mapview: Interactive Viewing of Spatial Data in R. 2023. Available: <https://github.com/r-spatial/mapview>) from the Reference list of our first draft. This is because we have removed and updated the map in Figure 1a in order to address the copyright issue in response to the editor’s comment. We have not cited any retracted articles in our paper.

[Ref. 37] Hu C, Li W, Wang R, Li Y, Li W, Zhang T. Insect flight speed estimation analysis based on a full- polarization radar. Science China Information Sciences. 2018; 61(10):1–3. https://doi.org/10.1007/s11432- 018-9484-2

[Ref. 43] Ulaby FT, Wilson EA. Microwave attenuation properties of vegetation canopies. IEEE Transactions on Geoscience and Remote Sensing. 1985; GE-23(5):746–753. 10.1109/TGRS.1985.289393

[Ref. 44] Jia M, Tong L, Zhang Y, Chen Y. Multitemporal radar backscattering measurement of wheat fields using multifrequency (L, S, C, and X) and full-polarization. Radio Science. 2013; 48:471–481. 10.1002/rds.20048

[Ref. 45] Gómez-Pérez P, Cuiñas I. Degradation of ground-based RADAR performance due to vegetation cover. Measurement. 2016; 92:230–235. https://doi.org/10.1016/j.measurement.2016.06.028.

[Ref. 46] Taylor PD, Brzustowski JM, Matkovich C, Peckford ML, Wilson D. radR: an open-source platform for acquiring and analysing data on biological targets observed by surveillance radar. BMC ecology. 2010; 10(1):22. https://doi.org/10.1186/1472-6785-10-22

[Ref. 47] Stepanian PM, Chilson PB, Kelly JF. An introduction to radar image processing in ecology. Methods in Ecology and Evolution. 2014; 5(8):730–738.  <https://doi.org/10.1111/2041-210X.12214>

**Author revision regarding Table format:** We have realized that we had placed the legend for Table 2 in the incorrect position. We regret this oversight. In this revised version, we have moved the legend below the table (p. 12, lines 268-269), in accordance with the journal’s guidelines.

**Author’s responses to Reviewer’s comments**

**Reviewer #1**

The authors have responded promptly and thoroughly to each of the reviewers’ comments: they provided detailed explanations for key revisions, supplemented necessary supporting data where requested, and adjusted the manuscript structure to enhance readability, all of which demonstrate a strong commitment to improving the work’s academic quality. As such, the manuscript, in its current form, is acceptable for publication.

**Author response**: Thank you for accepting our revisions. Again, we would like to express our gratitude for your time and effort in improving our paper.

**Reviewer #2**

**Reviewer comments:**

The revised manuscript demonstrates clear improvements in structure, transparency, and methodological explanation. The authors have carefully addressed many of the initial reviewer concerns by (1) introducing a dedicated Limitations subsection, (2) elaborating the radar echo extraction procedures, (3) clarifying statistical modeling decisions, (4) expanding discussion of clutter reduction, and (5) improving presentation and data accessibility.

The manuscript now reads clearly and is scientifically valuable as an empirical validation of a marine radar for bat detection. However, several key issues remain that require additional attention before final acceptance.

**Author response**: We appreciate your acceptance of our manuscript revisions. We would also like to thank you for your comments and suggestions for improving our manuscript. In the following sections, you will find our responses to each of your comments. All page numbers and line numbers refer to the revised manuscript file (Revised Manuscript with Track Changes) with tracked changes.

**Major Comments:**

**1. Robustness of manual echo identification**

Although the authors have added justification for manual selection based on flight direction and speed comparisons, the classification remains subjective. Only 25 out of 386 tracks were confirmed via ornithodolite matching, which may limit confidence in generalizing detection probabilities.

- Add a short sensitivity or validation analysis (e.g., varying inclusion thresholds for ground speed or track length) to show how detection probability estimates change under different selection criteria.
- If reanalysis is not feasible, state explicitly that the dataset’s small size and manual selection are limitations that may affect model generalizability.

**Author response**: We appreciate the reviewer’s comments on this point. We agree that this is an important point. In accordance with the reviewer’s comments, we have conducted a validation analysis by varying inclusion thresholds for ground-speed. These thresholds were determined based on the ground-speed of bats recorded by ornithodolite. We have established the following thresholds to filter radar tracks: 1) minimum–maximum (n = 384), 2) 10–90 percentiles (n = 352), and 3) 25–75 percentiles (n = 218) of bat’s ground-speed. Using these data, we have modeled the effect of distance on the probability of echo detection. In addition, two models were included in the validation: one that included all radar tracks (n = 386), and one that included tracks via ornithodolite matching (n = 25). We have reported the results of this validation in the supplementary materials (S3 Fig and S6 Table), and added the following text in the Statistical analysis subsection. We hope that our additional analysis adequately addresses your concern.

[p. 10, lines 236-241] Additionally, we examined how detection probability estimates change with a varying number of included radar tracks. To do so, we modeled the probability of echo detection using subsets of radar tracks filtered by thresholds determined based on the bat’s ground-speed (S6 Table). The results showed that the effect of distance on detection probability follows a similar pattern across models (S3 Fig, S6 Table). Although the small sample size and manual selection of radar tracks may affect the generalizability of the model, for this study’s GLMM, we used radar tracks extracted by matching bat trajectories recorded by ornithodolite.

**2. GLMM model interpretation and reporting**

The inclusion of odds ratios and 95% confidence intervals is appreciated, but practical interpretation is still limited.

- Report the odds ratio per 100 m increase in distance, or present a predicted probability curve (with 95% confidence bands) to illustrate detection decline with range.
- Include model diagnostics such as the random effect variance, sample size, and goodness-of-fit indices (AIC, R², or AUC).
- Consider including the post-hoc power analysis as supplementary material with a brief caveat about its interpretational limits.

We appreciate your advice on this point. In accordance with the reviewer’s suggestions, we have addressed each comment as follows:

**Author response 1**: We believe this point has been addressed in Fig 5 (positions in the main text: p. 11, line 263 and p. 12, line 271), which illustrates a predicted probability curve over distance with 95% confidence interval. We hope that the figure addresses your comment.

**Author response 2**: We have included the random effect variance and sample size in Table 2 (p. 12), R^2^ and AIC in the text of the Results section (p. 11, line 260).

**Author response 3**: We agree with the reviewer’s comment and have included the result of the post-hoc power analysis in the supplementary material, along with text explaining its limitations (S1 File). We have also added the following text in the Statistical analysis subsection:

[p. 10, lines 234-236] “Due to the small number of identified bat tracks (n = 25), we checked the statistical power of the model with the current sample size by conducting a post-hoc power analysis (S1 File).”

**3. Correlation and variable selection clarification**

The manuscript mentions a “Spearman’s rank correlation: r² = –0.71,” which appears to be a notation error.

- Replace “r²” with Spearman’s ρ (rho) to avoid confusion.
- Provide the scatterplot of distance vs. elevation angle in the supplementary materials.
- Briefly discuss whether orthogonalization or residualization could allow assessment of altitude effects independent of distance.

Thank you for pointing this out. In accordance with the reviewer’s comments, we have made revisions as follows:

**Author response 1**: We have replaced “r^2^” with “Spearman’s rho” in the text (p. 10, line 227).

**Author response 2**: We have included the scatterplot of distance and elevation angle in the supplementary materials (S2 Fig, positions in the main text: p. 10, line 228).

**Author response 3**: We have added the following text in the Statistical analysis subsection. For clarification, we have also reported the result of a GLMM, which was analyzed the effect of elevation angle on detection probability in S5 Table.

[p. 10, lines 228-231] “While orthogonalization or residualization may allow simultaneous analysis for both variables, this approach was not used in this study. This is because it becomes difficult to interpret model estimates after applying these techniques. Additionally, elevation angle alone did not significantly affect the probability of echo detection (S5 Table).”

**Summary Recommendation**

The manuscript has improved substantially and now meets most of the journal’s scientific standards. The remaining issues are primarily technical and analytical refinements to enhance transparency and interpretability.

**Recommendation:** Minor Revision (acceptable after clarification and supplementary additions).

Again, thank you for giving us the opportunity to strengthen our manuscript with your comments and suggestions. We have worked hard to incorporate your comments and hope that these revisions are sufficient to make our submission suitable for publication in PLOS ONE.

Sincerely,

Yoichi Kawaguchi

Graduate School of Technology, Industrial, and Social Sciences, Tokushima University

2-1 Minamijosanjima-cho, Tokushima, Japan

TEL: +81-88-656-9025

E-mail: kawaguchi@ce.tokushima-u.ac.jp
