# Supplementary material for: Detection performance of an X-band marine radar system for free-flying Asian particolored bats (Vespertilio sinensis)
Source: PLoS One. 2025 Nov 20;20(11):e0337422. doi: 10.1371/journal.pone.0337422 (PMC12633934; doi:10.1371/journal.pone.0337422)
Supplement: Attachment — Submitted filename: Reviewer_comments_declet_2.docx [file pone.0337422.s016.docx]

**Reviewer Comments:**

1. **Robustness of Manual Echo Identification:**
   - **Comment:** The manual identification of radar echoes as bat flights remains subjective. Only 25 out of 386 tracks were verified using the ornithodolite, which may limit the generalizability of detection probabilities.
   - **Suggested Actions:** The authors should conduct a sensitivity analysis by varying the inclusion thresholds for parameters like ground speed and track length to examine how detection probability estimates change under different criteria. If reanalysis is not feasible, they should explicitly mention the small sample size and manual selection as limitations that may affect model generalizability.
2. **GLMM Model Interpretation and Reporting:**
   - **Comment:** The inclusion of odds ratios and 95% confidence intervals is useful, but the interpretation of the results could be more practical.
   - **Suggested Actions:**
     - Report the odds ratio per 100 m increase in distance or provide a predicted probability curve (with 95% confidence bands) to illustrate detection decline with range.
     - Include model diagnostics such as random effect variance, sample size, and goodness-of-fit indices (AIC, R², or AUC).
     - Consider adding the post-hoc power analysis as supplementary material, with a caveat about its limits.
3. **Correlation and Variable Selection Clarification:**
   - **Comment:** The manuscript mentions "Spearman’s rank correlation: r² = –0.71," which appears to be a notation error. The correct term is Spearman’s ρ (rho).
   - **Suggested Actions:**
     - Replace "r²" with "Spearman’s ρ" to avoid confusion.
     - Provide the scatterplot of distance vs. elevation angle in supplementary materials.
     - Discuss whether orthogonalization or residualization could assess altitude effects independent of distance.

**Summary Recommendation:**

- **Minor Revision:** Acceptable after addressing the clarifications and statistical improvements.
