# Supplementary material for: Detection performance of an X-band marine radar system for free-flying Asian particolored bats (Vespertilio sinensis)
Source: PLoS One. 2025 Nov 20;20(11):e0337422. doi: 10.1371/journal.pone.0337422 (PMC12633934; doi:10.1371/journal.pone.0337422)
Supplement: Attachment — Submitted filename: Reviewer comments.docx [file pone.0337422.s014.docx]

**Reviewer comments:**

The revised manuscript demonstrates clear improvements in structure, transparency, and methodological explanation. The authors have carefully addressed many of the initial reviewer concerns by (1) introducing a dedicated Limitations subsection, (2) elaborating the radar echo extraction procedures, (3) clarifying statistical modeling decisions, (4) expanding discussion of clutter reduction, and (5) improving presentation and data accessibility.

The manuscript now reads clearly and is scientifically valuable as an empirical validation of a marine radar for bat detection. However, several key issues remain that require additional attention before final acceptance.

**Major Comments:**

**1. Robustness of manual echo identification**

Although the authors have added justification for manual selection based on flight direction and speed comparisons, the classification remains subjective. Only 25 out of 386 tracks were confirmed via ornithodolite matching, which may limit confidence in generalizing detection probabilities.

- Add a short sensitivity or validation analysis (e.g., varying inclusion thresholds for ground speed or track length) to show how detection probability estimates change under different selection criteria.
- If reanalysis is not feasible, state explicitly that the dataset’s small size and manual selection are limitations that may affect model generalizability.

**2. GLMM model interpretation and reporting**

The inclusion of odds ratios and 95% confidence intervals is appreciated, but practical interpretation is still limited.

- Report the odds ratio per 100 m increase in distance, or present a predicted probability curve (with 95% confidence bands) to illustrate detection decline with range.
- Include model diagnostics such as the random effect variance, sample size, and goodness-of-fit indices (AIC, R², or AUC).
- Consider including the post-hoc power analysis as supplementary material with a brief caveat about its interpretational limits.

**3. Correlation and variable selection clarification**

The manuscript mentions a “Spearman’s rank correlation: r² = –0.71,” which appears to be a notation error.

- Replace “r²” with Spearman’s ρ (rho) to avoid confusion.
- Provide the scatterplot of distance vs. elevation angle in the supplementary materials.
- Briefly discuss whether orthogonalization or residualization could allow assessment of altitude effects independent of distance.

**Summary Recommendation**

The manuscript has improved substantially and now meets most of the journal’s scientific standards. The remaining issues are primarily technical and analytical refinements to enhance transparency and interpretability.

**Recommendation:** Minor Revision (acceptable after clarification and supplementary additions).
